# Multicomponent Nutritional Support for Children with Autism Spectrum Disorder: An Exploratory Pilot Study in Vietnam

**DOI:** 10.3390/pediatric18040097

**Published:** 2026-07-13

**Authors:** Ngoc Dieu Thi Phan, Toan Thi Thanh Do, Tuan Van Nguyen, Ngoc Bao Trinh, Huy Gia Ngo, Hoa Thi Ho, Tam Thi Thanh Le, Hiep Tri Ngo

**Affiliations:** 1 School of Preventive Medicine and Public Health, Hanoi Medical University, 1 Ton That Tung Street, Khuong Thuong Ward, Dong Da District, Hanoi 100000, Vietnam; nguyenngoc.vmu@gmail.com (N.D.T.P.); huygiango3001@gmail.com (H.G.N.); 2 Faculty of Public Health, Vinh Medical University, 161 Nguyen Phong Sac Street, Hung Dung Ward, Vinh City 430000, Vietnam; drhieplinh@gmail.com; 3 Faculty of Medicine, Vinh Medical University, 161 Nguyen Phong Sac Street, Hung Dung Ward, Vinh City 430000, Vietnam; tuanminh1975@gmail.com (T.V.N.); thanhtam@vmu.edu.vn (T.T.T.L.); 4 Institute of Nutrition Research and Development, 48B Tang Bat Ho Street, Hai Ba Trung Ward, Hanoi 100000, Vietnam; trinhbaongocdd1967@gmail.com (N.B.T.); hohoa1905@gmail.com (H.T.H.)

**Keywords:** Autism Spectrum Disorder, nutritional intervention, nutrition counseling, micronutrients, growth, dietary intake

## Abstract

Background: Children with Autism Spectrum Disorder (ASD) frequently exhibit severe food selectivity and micronutrient deficiencies, impacting growth and nutritional status. Evidence on integrated nutritional interventions in resource-constrained settings remains limited. Objective: To assess the feasibility and preliminary pre–post changes associated with a 12-week multicomponent nutritional intervention among Vietnamese children with ASD. Methods: In this exploratory single-arm pilot study, 56 children with ASD (mean age 59.0 ± 22.3 months; 80.4% male) were recruited from five community centers in Nghe An Province, Vietnam. The intervention comprised caregiver nutrition education, individualized dietary counseling, and daily multi-micronutrient supplementation. Anthropometric indicators, biochemical markers, feeding behaviors, and dietary intake were assessed at baseline and after 12 weeks. Pre–post changes were evaluated using paired statistical tests, and multivariable linear regression examined factors associated with growth response. Results: The study achieved a 100% completion rate, with all 56 recruited participants finishing the 12-week intervention and all scheduled follow-up assessments. Among children < 60 months, mean Weight-for-Age Z-score (WAZ) increased from −0.66 to −0.28 and mean Height-for-Age Z-score (HAZ) from −1.18 to −0.97 (*p* < 0.001). For children older than 60 months, mean HAZ increased from −0.87 to −0.58 (*p* < 0.001) and Body Mass Index-for-Age Z-score (BAZ) from −0.20 to 0.06 (*p* = 0.006). Significant increases occurred in serum zinc (10.29 to 11.72 µmol/L; *p* = 0.001), ferritin (31.74 to 34.79 ng/mL; *p* = 0.001), and hemoglobin (122.73 to 124.77 g/L; *p* = 0.002), while albumin remained unchanged. Concurrent improvements were observed in feeding behaviors and nutrient-dense food intake. Regression analysis indicated that lower baseline anthropometric status was significantly associated with greater gains in WAZ and HAZ. Conclusions: This community-based multicomponent intervention was feasible and associated with short-term improvements in feeding behaviors, dietary intake, selected biomarkers, and growth measures in children with ASD, supporting further evaluation in randomized controlled trials.

## 1. Introduction

Autism Spectrum Disorder (ASD) is a neurodevelopmental condition characterized by persistent deficits in social communication and restricted patterns of behavior. The prevalence of ASD has increased substantially worldwide in recent decades, making it an important public health concern. Beyond the core behavioral manifestations, children with ASD often experience a range of physical health challenges, including nutritional problems that may adversely affect growth, neurodevelopment, cognitive functioning, and overall quality of life [1,2].

Nutritional status among children with ASD is often driven by food selectivity, sensory sensitivities, restricted dietary variety, and feeding difficulties. Previous studies have consistently reported inadequate intake of fruits, vegetables, and protein-rich foods, along with a preference for highly processed, energy-dense products. Consequently, children with ASD are at risk of both growth impairment and micronutrient deficiencies [3,4,5], particularly deficiencies of iron, zinc, vitamin D, and B vitamins [6]. These nutritional inadequacies may further compromise physical development, immune function, and neurodevelopment outcomes.

Given the multifactorial nature of nutritional problems in ASD, nutritional interventions have increasingly been recognized as a potentially important component of comprehensive care. Strategies such as nutrition education, individualized dietary counseling, feeding behavior modification, and micronutrient supplementation have been proposed to improve dietary quality and nutritional status. However, current evidence remains limited and heterogeneous. Most published studies have focused on describing nutritional status rather than evaluating intervention effectiveness. Furthermore, few studies have examined multicomponent interventions that simultaneously address feeding behaviors, dietary quality, and micronutrient deficiencies. Evidence from low and middle-income countries is particularly scarce.

Vietnam is currently experiencing a rapid nutritional transition, with undernutrition and micronutrient deficiencies persisting alongside changing dietary patterns. Despite the increasing number of children diagnosed with ASD, evidence regarding nutritional interventions for this population remains limited. To our knowledge, no community-based study in Vietnam has comprehensively evaluated the effects of an integrated nutritional intervention combining caregiver education, individualized dietary counseling, and micronutrient supplementation on growth outcomes, dietary intake, feeding behaviors, and biochemical nutritional indicators among children with ASD.

Therefore, this study aimed to evaluate the feasibility and potential effects of a 3-month multicomponent nutritional intervention among Vietnamese children with ASD. Specifically, we assessed changes in anthropometric indicators and biochemical nutritional markers as primary outcomes, and dietary intake patterns and feeding behaviors as secondary outcomes. We hypothesized that participation in the intervention would be associated with improvements in dietary quality, feeding behaviors, nutritional biomarkers, and growth indicators.

## 2. Methods

### 2.1. Study Design and Setting

This prospective single-arm pilot intervention study was conducted between July 2024 and May 2025 among children with ASD attending five community-based ASD intervention centers in Nghe An Province, Vietnam.

### 2.2. Participants

Children were eligible if they:

(1) Had a clinical diagnosis of ASD established by a specialized pediatric hospital in Vietnam;

(2) Were enrolled in one of the participating intervention centers;

(3) Had a primary caregiver willing to participate in nutrition counseling sessions and provide informed consent.

Children were excluded if they:

(1) Had acute infections or severe chronic diseases affecting nutritional status;

(2) Had severe gastrointestinal disorders associated with malabsorption;

(3) Were receiving high-dose micronutrient supplementation or participating in other nutrition-related intervention studies.

Participants were recruited through convenience sampling from five participating centers. Written informed consent was obtained from all caregivers before enrollment.

### 2.3. Sampling Methods

Participants were recruited using a non-probability convenience sampling approach through direct referrals from the five participating ASD intervention centers. Given the exploratory nature of this pre–post pilot study, the primary objectives were to assess feasibility and to obtain preliminary estimates of change in nutritional and behavioral outcomes, rather than to formally test hypotheses.

In line with methodological recommendations for pilot interventions, we aimed to enroll at least 30 children to ensure stable variance estimates and to inform planning for a future randomized controlled trial. The final sample of 56 participants exceeded this minimum target. It was deemed sufficient to: (1) provide reasonably precise estimates of within-subject changes in anthropometric z-scores and biochemical markers over 12 weeks, and (2) allow exploratory multivariable modeling of predictors of growth response while maintaining an acceptable events-per-variable ratio. This sample size should therefore be interpreted as pragmatic and adequate for a feasibility pilot, rather than as the result of a formal power calculation for a specific primary endpoint.

### 2.4. Intervention

The intervention consisted of three integrated components delivered over 12 weeks:

#### 2.4.1. Caregiver Nutrition Education

At baseline, caregivers attended a standardized group education session conducted by trained nutrition professionals. The session covered nutritional requirements for children with ASD, healthy meal planning, management of feeding difficulties, and strategies to improve dietary diversity.

#### 2.4.2. Individualized Dietary Counseling

Caregivers received individualized dietary counseling delivered by trained nutritionists throughout the 12-week intervention period. Each counseling session lasted approximately 30–45 min and was conducted at baseline, 1 month, and 2 months after enrollment, with additional sessions provided as needed to address the child’s feeding difficulties and caregiver support needs.

Dietary recommendations were developed based on age-specific nutritional requirements and tailored to the behavioral characteristics of children with ASD. Counseling emphasized adequate energy intake, balanced macronutrient consumption, dietary diversity, and increased consumption of nutrient-dense foods, including lean meats, fish, eggs, dairy products, legumes, fruits, and vegetables. Particular attention was given to food sources rich in iron, zinc, vitamin D, and B vitamins. Caregivers were also encouraged to reduce children’s consumption of ultra-processed foods, sugar-sweetened beverages, sweets, and fried foods and replace them with healthier alternatives.

To address food selectivity and sensory-related feeding difficulties, individualized behavioral feeding strategies were implemented, including gradual exposure to unfamiliar foods, texture modification, structured meal schedules, and positive reinforcement during mealtime. Caregivers maintained daily food diaries, which were reviewed during follow-up visits to guide personalized dietary adjustments.

#### 2.4.3. Multi-Micronutrient Supplementation

Given the high prevalence of restricted dietary patterns and micronutrient inadequacies among children with ASD, participants received a daily multi-micronutrient supplement throughout the 12-week intervention period as an adjunct to dietary counseling.

The supplement used in this study was a commercially available liquid multi-micronutrient formulation (Anni Baby Multi; AN CARE Pharma, Hanoi, Vietnam). The Vietnam Food Administration has granted the product a Certificate of Registered Product Declaration—Ministry of Health (Reference No: 4571/2022/ĐKSP). This formulation was selected because it provides several micronutrients commonly reported as inadequate in children with ASD, including zinc, vitamin D, and B vitamins. Detailed nutrient composition is provided in Appendix A Table A1.

The supplement was distributed monthly to caregivers and administered according to age-specific manufacturer recommendations. Caregivers were instructed to provide the supplement once daily and record administration in a daily logbook.

Adherence was monitored through caregiver records and monthly supplement audits, including the return and inspection of supplement containers during follow-up visits. Caregivers were also instructed to report any adverse events throughout the intervention period. No serious adverse events related to supplementation were reported.

### 2.5. Outcome Measures

All outcome assessments were performed at baseline and at the end of the 12-week intervention period. The study primarily aimed to evaluate changes in nutritional status and micronutrient profiles following participation in the intervention. Accordingly, the primary outcomes included anthropometric indicators (WAZ and HAZ) and biochemical nutritional markers, including serum zinc, ferritin, hemoglobin, and albumin concentrations.

Secondary outcomes focused on potential behavioral and dietary changes associated with the intervention. These included feeding behaviors, food selectivity, dietary intake, and food group consumption patterns. Dietary intake was assessed using repeated 24 h dietary recalls and a food frequency questionnaire, while feeding behaviors were evaluated using a standardized caregiver-reported assessment tool.

#### 2.5.1. Anthropometric Assessment

Body weight was measured to the nearest 0.1 kg using a calibrated digital scale (Tanita WB-3000, Tanita Corporation, Tokyo, Japan). Height was recorded to the nearest 0.1 cm using a portable stadiometer (Seca 206, Seca GmbH & Co. KG, Hamburg, Germany). To enhance the quality of these measurements and minimize technical errors in Standardized Conditions, all anthropometric evaluations were consistently performed in the morning hours, with the children wearing light clothing and no shoes. Measurements were performed by trained personnel in accordance with standardized procedures.

For children younger than 60 months, WAZ, HAZ, and Weight-for-Height Z-score (WHZ) were calculated. For children aged 60 months and older, HAZ and BMI-for-Age Z-score (BAZ) were calculated according to the WHO Child Growth Standards [7,8]. Z-scores were computed using WHO Anthro software (version 3.2.2; World Health Organization, Geneva, Switzerland) for children younger than 60 months and WHO AnthroPlus software (version 1.0.4; World Health Organization, Geneva, Switzerland) for older children. 

#### 2.5.2. Biochemical Assessment

Venous blood samples were collected at baseline and after completion of the 12-week intervention to evaluate changes in micronutrient and hematological status. Blood collection was performed on-site at the participating intervention centers by trained healthcare professionals from Nghe An Obstetrics and Pediatrics Hospital following standardized biosafety and infection-control procedures. To minimize distress and facilitate cooperation, primary caregivers remained present during sample collection.

Following collection, samples were transported under controlled conditions to the hospital laboratory for immediate processing and analysis. To reduce measurement bias, all laboratory personnel were blinded to study objectives and assessment time points. Samples were de-identified and labeled using unique numerical codes before laboratory analysis.

The biochemical indicators assessed included hemoglobin, serum ferritin, serum zinc, and serum albumin. Hemoglobin was measured using the cyanmethemoglobin method on an XN-2000 Automated Hematology Analyzer (Sysmex Corporation, Kobe, Japan). Serum albumin and serum zinc were measured on an AU680 Clinical Chemistry Analyzer (Beckman Coulter, Inc., Brea, CA, USA), and serum ferritin was measured on a DxI 800 Access Immunoassay System (Beckman Coulter, Inc., Brea, CA, USA). Anemia was defined as hemoglobin concentrations below 110 g/L in children younger than five years and below 115 g/L in children aged 5–11 years. Serum zinc concentrations below 9.9 μmol/L were classified as zinc deficiency. Serum ferritin concentrations below 12 ng/mL in children under five years and below 15 ng/mL in children aged 5–19 years were considered indicative of depleted iron stores. Serum albumin concentrations above 34 g/L were considered within the normal range [9].

### 2.6. Adherence Monitoring and Safety Assessment

Adherence to the intervention was monitored throughout the 12-week study period using a structured supervision framework involving caregivers, teachers, and study personnel. Compliance with multi-micronutrient supplementation was documented daily by caregivers or teachers, depending on the site of administration. Monthly follow-up visits included supplement audits, during which administration records were reviewed, and returned supplement containers were inspected to verify compliance.

Adherence to dietary recommendations was monitored through caregiver-maintained food diaries, which were reviewed every two weeks by study nutritionists. These reviews were used to assess dietary diversity, identify feeding challenges, and provide individualized feedback to support implementation of dietary recommendations. Attendance at nutrition counseling sessions was also recorded throughout the intervention period. Compliance with the counseling component was defined as participation in at least three individualized counseling sessions during the study period.

Safety monitoring was conducted concurrently with adherence assessment. Caregivers were instructed to document and report any adverse events or unexpected behavioral changes throughout the intervention period. Information regarding gastrointestinal symptoms, allergic reactions, and other health concerns was reviewed during follow-up visits. No serious adverse events related to the intervention were reported.

The study achieved complete participant retention and intervention adherence throughout the 12 weeks. All enrolled participants completed follow-up assessments, attended the required counseling sessions, and received the full supplementation protocol.

### 2.7. Statistical Analysis

Data were analyzed using R version 4.2.1 (R Foundation for Statistical Computing, Vienna, Austria). Descriptive statistics were used to summarize participant characteristics, intervention adherence, and study outcomes. Categorical variables are presented as frequencies and percentages, whereas continuous variables are reported as means and standard deviations (SD).

As this was an exploratory pilot study, the analyses were primarily intended to describe feasibility and estimate the direction and magnitude of pre–post changes across study outcomes rather than to draw definitive causal inferences. Continuous outcomes, including anthropometric indicators, biochemical markers, and nutrient intake variables, were compared between baseline and post-intervention assessments using paired statistical tests, as appropriate for the data distribution. Ordered feeding-behavior variables were analyzed using appropriate paired comparisons for ordinal data.

Mean changes and 95% confidence intervals were calculated for key continuous outcomes to provide preliminary estimates for future trial planning. Feasibility outcomes, including retention, adherence to supplementation and counseling, completion of follow-up assessments, and adverse events, were summarized descriptively.

To explore factors associated with growth response during follow-up, multivariable linear regression models were fitted, with changes in anthropometric z-scores as dependent variables. Candidate predictors included baseline anthropometric status, age, and selected changes in behavioral, dietary, and biochemical indicators. These models were prespecified as exploratory and should be interpreted as hypothesis-generating. Statistical significance was defined as a two-sided *p*-value < 0.05.

## 3. Results

### 3.1. Participant Recruitment and Baseline Characteristics

#### 3.1.1. Feasibility Outcomes

A total of 56 children with ASD were enrolled and initiated the 12-week intervention. All participants completed the intervention and post-intervention assessments, resulting in a 100% retention rate. Compliance with the intervention was high, with all caregivers attending the required counseling sessions and completing the supplementation protocol according to study procedures. Adherence to dietary recommendations was monitored throughout the intervention using food diaries and follow-up consultations. Monthly supplement audits and caregiver records indicated excellent compliance with micronutrient supplementation. No serious adverse events or intervention-related safety concerns were reported during the study period.

#### 3.1.2. Participant Characteristics

The study included 56 children with ASD, with a mean age of 59.0 ± 22.3 months (Table 1). Most participants were male (80.4%), reflecting the known sex distribution of ASD. Approximately half of the participants were younger than 60 months, and the majority of caregivers had completed secondary or university education.

### 3.2. Anthropometric Status and Growth Indicators

The 12-week nutritional intervention was associated with measurable improvements in growth indicators across both age cohorts (Table 2). Among children younger than 60 months, the median WAZ increased by 0.27 standard deviation units (Q1–Q3: 0.14 to 0.55), while the median HAZ increased by 0.21 standard deviation units (Q1–Q3: 0.05 to 0.36) over the 12-week intervention period. In contrast, changes in WHZ did not reach statistical significance. Among children aged 60 months and older, the median HAZ increased by 0.26 standard deviation units (Q1–Q3: 0.12 to 0.34), and the median BAZ increased by 0.26 standard deviation units (Q1–Q3: −0.03 to 0.57).

### 3.3. Changes in Feeding Behaviors, Dietary Patterns, and Nutrient Intake

Structured assessment of mealtime behaviors demonstrated substantial improvements in feeding efficiency following the intervention (Table 3). The prevalence of prolonged food-holding behaviors declined markedly, with a clear shift toward children no longer holding food in the mouth. Meal duration profiles improved in parallel, as extended mealtimes became less common and shorter timeframes for completion became more common. Indicators of mealtime progression also shifted favorably, with the majority of children transitioning to rapid, uninterrupted eating from start to finish.

Furthermore, core maladaptive eating behaviors showed similarly robust improvement. High dietary selectivity decreased substantially, accompanied by a pronounced increase in the proportion of children consuming a more varied range of foods. Sensory-based food refusal became less frequent, with a notable increase in children who demonstrated occasional or no sensory aversion. Reports of oppositional and avoidance behaviors during meals declined sharply. At the same time, the proportion of children exhibiting full mealtime compliance increased, with the majority of the cohort in full compliance by the end of the intervention period.

**Figure 1:**
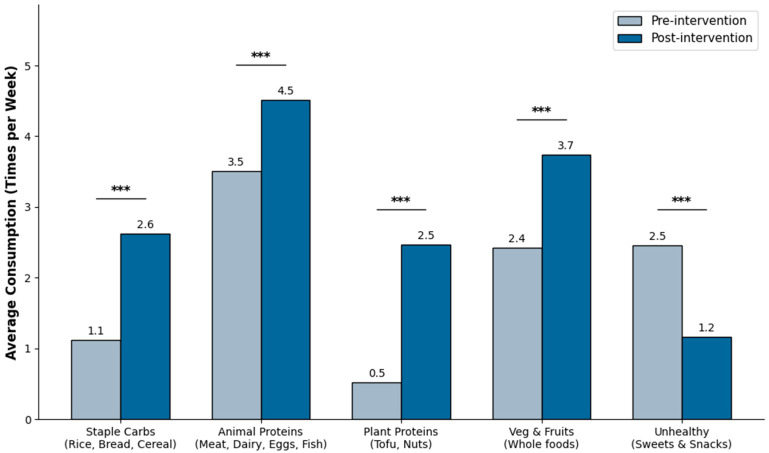
Shifts in Weekly Food Group Consumption. Note: *** indicates that *p* < 0.001.

Following the 3-month intervention, a highly statistically significant shift toward healthier eating patterns emerged, accompanied by a sharp decline in unhealthy choices (Figure 1). Whole vegetables and fruits rose from a baseline mean of 2.42 to 3.74 times per week, while animal-source proteins advanced from 3.51 to 4.51 times per week. Plant-based proteins (tofu and nuts) demonstrated the most dramatic relative surge, expanding nearly fivefold from 0.52 to 2.47 times per week. Conversely, unhealthy sweets and snacks more than halved, dropping from 2.46 to 1.16 times per week. Staple carbohydrates experienced a more moderate upward shift, increasing from a baseline mean of 1.12 to 2.62 times per week.

**Figure 2:**
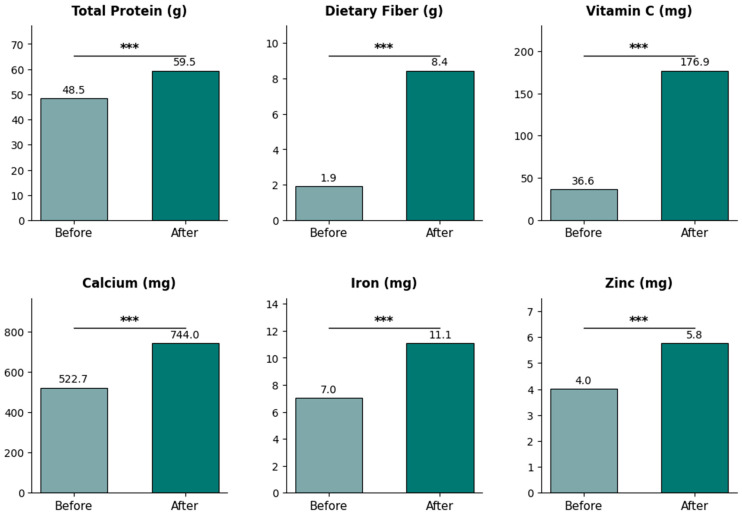
Absolute Changes in Daily Nutrient Intake. Note: *** indicates *p* < 0.001.

Daily nutrient intake profiles showed a broad, consistent, and significant upward shift over the 12-week intervention period (Figure 2). Overall dietary exposure increased substantially across all assessed macro- and micronutrients. The most pronounced relative gains were observed for vitamin C and dietary fiber, both of which increased several-fold compared with baseline levels. Parallel improvements were noted for key macronutrients and minerals. Total protein intake increased markedly, accompanied by substantial gains in calcium consumption. Intakes of essential trace minerals, including iron and zinc, also rose consistently across the cohort.

### 3.4. Changes in Biochemical Parameters

Several biochemical indicators showed favorable changes following the 12-week intervention (Table 4). Mean serum zinc concentrations increased from 10.29 to 11.72 µmol/L, representing the largest observed change among the biochemical outcomes. Mean ferritin concentrations also increased modestly, while hemoglobin concentrations showed a small but statistically significant increase. In contrast, serum albumin concentrations remained stable throughout the intervention period. Overall, these findings suggest improvements in selected micronutrient and hematological indicators during the intervention period, whereas markers of general protein status showed little change.

### 3.5. Predictors of Growth Outcomes

Exploratory regression analyses suggested that baseline anthropometric status was associated with the magnitude of subsequent anthropometric change during the intervention period (Table 5). Children with lower baseline WAZ and HAZ tended to demonstrate larger improvements in growth indicators over follow-up. No significant associations were observed for changes in feeding behavior, protein intake, or serum zinc concentrations. These findings should be interpreted cautiously, given the exploratory nature of the analyses and the absence of a control group.

## 4. Discussion

In this 12-week exploratory pre–post study, a multicomponent nutritional intervention was associated with improvements across anthropometric, behavioral, and biochemical domains in Vietnamese children with ASD. The baseline cohort exhibited a pronounced male predominance (4:1), consistent with reported sex ratios in autism epidemiology [10,11]. Following the intervention, participants demonstrated significant linear growth and weight gain, microchemical recovery, and a marked reduction in maladaptive feeding behaviors, illustrating the potential benefit of a community-embedded, caregiver-mediated approach.

### 4.1. Behavior as the Foundation for Nutritional Change in Children with ASD

The substantial transition toward dietary adequacy observed in this cohort suggests a fundamental shift in behavioral rigidity rather than mere caloric manipulation. For children with ASD, abnormal eating behaviors such as sensory aversions and severe food selectivity represent the primary barriers to nutritional adequacy [12,13,14,15]. Our findings underscore that addressing maladaptive feeding behaviors is a prerequisite for nutritional recovery in ASD. Structured caregiver counseling appeared to reduce sensory-based refusal and food selectivity, thereby enabling both diversification of whole foods and consistent micronutrient supplementation [14].

This behavioral resolution aligns with broader evidence confirming that targeting parental feeding approaches can effectively overcome pediatric feeding aversions [16]. Also, primary caregivers function as the vital operational link between clinical recommendations and home-based execution [17]. Their nuanced, experiential understanding of the child’s specific sensory triggers allows for precise, adaptive implementation of dietary strategies during routine meals. Ultimately, these outcomes reinforce the clinical principle that nutritional and adherence gains in pediatric autism are not spontaneous but are actively driven by behavioral and environmental modifications facilitated by trained caregivers [17,18].

### 4.2. Differential Biochemical Responsiveness to the Intervention

The intervention yielded a divergent pattern of biochemical changes, characterized by measurable enhancements in micronutrient and hematological markers (serum zinc, ferritin, and hemoglobin) alongside stagnant visceral protein status. This variance indicates that distinct biochemical indices exhibit differential short-term sensitivity within a 12-week timeframe. Because dietary counseling and multi-micronutrient supplementation were administered concurrently, the independent relative contributions of each component cannot be statistically isolated. Rather than supporting component-specific attribution, these findings highlight holistic, short-term microchemical responsiveness within a short time frame following a multi-component intervention.

### 4.3. Growth Dynamics and Baseline Nutritional Status

Multivariable linear regression modeling revealed that baseline nutritional status was the primary independent predictor of longitudinal growth trajectories. The strong inverse relationship observed between baseline HAZ/WAZ and subsequent somatic adjustments supports the physiological consensus that growth velocity accelerates dynamically upon the correction of prior nutritional deprivation [19]. The pattern of larger gains among children with lower baseline z-scores is consistent with physiological catch-up growth observed after alleviation of prior nutritional deficits. However, this interpretation remains tentative given the short follow-up and lack of a comparison group [19].

Conversely, variables such as age, dietary protein intake, and short-term fluctuations in serum zinc did not emerge as independent drivers of weight gain in the adjusted models. This statistical analysis suggests that, within brief intervention windows, somatic recovery is overwhelmingly determined by initial physiological reserves and catch-up dynamics rather than concurrent biochemical or dietary variability.

### 4.4. The Public Health Implications

From a public health perspective, these preliminary outcomes validate the feasibility and ecological validity of implementing integrated nutritional strategies within community-based specialized centers, thereby circumventing the need for overextended tertiary healthcare infrastructure [20,21]. In resource-limited settings undergoing rapid nutritional transitions, low-cost behavioral tactics such as structured food exposure offer a highly scalable alternative to expensive clinical therapies [16]. 

Furthermore, the profound restructuring of daily food consumption patterns underscores the value of institutional alignment, where center-based teachers and staff spend substantial daytime hours with the children, gaining firsthand insight into their mealtime behavioral triggers. However, our protocol noted an implementation asymmetry. While primary parental caregivers received intensive, individualized counseling, center-based staff were limited to baseline group education sessions and general instructional handbooks. This gap signals the need for a new public health approach in resource-limited settings. Instead of broad training, future models should offer focused education to center staff so they can help parents use consistent feeding strategies at home and in care facilities.

At the healthcare system level, embedding routine nutritional screenings directly into established community early intervention networks provides an alternative model for addressing localized vulnerabilities. Given the exploratory, single-group pilot nature of this trial, these outcomes constitute a preliminary proof of concept rather than a definitive policy directive. Nevertheless, these findings suggest that the preliminary data warrant systematic validation through larger randomized controlled trials to evaluate long-term scalability, cost-effectiveness, and health equity rigorously.

### 4.5. Strengths and Limitations

This study possesses several distinct strengths in evaluating an integrated, multicomponent nutritional strategy under real-world conditions across five community-based specialized centers. A key methodological advantage is the utilization of highly objective outcome measures, specifically synchronized anthropometric Z-scores and venous biochemical markers, which mitigated response bias and provided a reliable assessment of nutritional status. Additionally, the cohort exhibited high clinical homogeneity at enrollment, with all 56 participants free of confounding somatic or psychiatric comorbidities, whilst achieving 100% adherence and retention, underscoring the feasibility of this community-embedded pediatric framework.

Despite these strengths, several critical methodological limitations must be acknowledged. Primarily, the single-group pre–post design lacked a concurrent control group, making it impossible to rule out time-related confounding variables. Because dietary counseling and multi-micronutrient supplementation were implemented simultaneously, their independent effects could not be statistically isolated. This constraint was further compounded by the use of convenience sampling via institutional referrals, which introduced potential selection bias, as this cohort might be composed of highly motivated, university-educated caregivers who could be more amenable to clinical research. Moreover, the study’s small sample size (*n* = 56) limited statistical power, thereby raising the risk of both statistical Type I and Type II errors. Specifically, the risk of a Type I error is elevated because, in smaller cohorts, extreme individual variations or outliers can disproportionately skew the overall distribution, leading to statistically significant pre–post differences that may not reflect a true, stable population-level intervention effect. Conversely, the study was vulnerable to Type II errors when evaluating subgroup differences. Due to the reduced sample sizes within these stratified subgroups, the statistical analysis lacked sufficient power to detect subtle yet clinically meaningful differences, potentially failing to identify true localized therapeutic benefits.

Furthermore, the intervention’s 3-month follow-up period remains insufficient to evaluate long-term dietary sustainability, structural behavioral changes, or permanent somatic development. Lastly, the study’s generalizability is limited by its focus on a single Vietnamese province with specific cultural and dietary contexts, and by the pronounced male predominance (80.4%), which limits extrapolation to other ethnicities, geographic regions, and female pediatric autism populations.

## 5. Conclusions

The present pilot study suggests that a community-based nutritional intervention incorporating caregiver education, individualized dietary counseling, and multi-micronutrient supplementation is feasible and well accepted among children with ASD and their caregivers in Vietnam. Favorable changes were observed across feeding behaviors, dietary patterns, biochemical nutritional indicators, and growth outcomes during the intervention period.

These preliminary findings support the potential value of integrating nutritional support into community-based ASD services. However, adequately powered randomized controlled trials are required to establish effectiveness and inform future implementation strategies.

## Figures and Tables

**Table 1:** Baseline Demographic and Clinical Characteristics of the Study Participants (*n* = 56).

Characteristic	n (%)
Gender	
Male	45 (80.4)
Female	11 (19.6)
Overall Age (months)	
Mean (SD)	59.0 (22.3)
Median	63.5
<60 months	26 (46.4)
≥60 months	30 (53.6)
Range (Min–Max)	23.8–119.5
Age Subgroups (months)	
<60 months	26 (46.4)
Mean (SD)	45.2 (10.3)
Median	45.7
Range (Min–Max)	23.8–59.3
≥60 months	30 (53.6)
Mean (SD)	79.7 (16.0)
Median	74.2
Range (Min–Max)	62.1–119.5
Age at ASD diagnosis (months)	
12–18 months	8 (14.3)
19–24 months	22 (39.3)
>24 months	26 (46.4)
Mean (SD)	27.0 (8.9)
Range	12–60
Caregiver Education Level	
Lower Secondary School	8 (14.3)
Upper Secondary School	19 (33.9)
University	29 (51.8)
Comorbidities (Other than ASD)	
No	56 (100.0)
Household Income per Capita	
Poor household status	1 (1.8)
1–<3 million VND/month	7 (12.5)
3–8 million VND/month	34 (60.7)
>8 million VND/month	14 (25.0)

**Table 2:** Changes in Anthropometric Z-scores Following 3-Month Intervention.

Age Group/Indicator	Pre-Intervention Median (Q1–Q3)	Post-Intervention Median (Q1–Q3)	Median Difference (Q1–Q3)	*p*-Value *
Children < 60 months (*n* = 26)				
Weight-for-Age (WAZ)	−0.91 (−1.13–0.21)	−0.45 (−0.86–0.33)	0.27 (0.14–0.55)	**<0.001**
Height-for-Age (HAZ)	−1.02 (−1.67–−0.52)	−0.94 (−1.45–−0.29)	0.21 (0.05–0.36)	**<0.001**
Weight-for-Height (WHZ)	−0.18 (−0.44–0.66)	−0.32 (−0.71–0.72)	−0.41 (−0.75–0.70)	0.77
Children ≥ 60 months (*n* = 30)				
Height-for-Age (HAZ)	−1.00 (−1.57–0.03)	−0.56 (−1.30–0.43)	0.26 (0.12–0.34)	**<0.001**
BMI-for-Age (BAZ)	−0.54 (−1.11–0.78)	−0.13 (−0.84–0.83)	0.26 (−0.03–0.57)	**0.006**

Note: * *p*-values were calculated using the Wilcoxon Signed-Rank Test for paired samples.

**Table 3:** Changes in Dietary Quality and Feeding Behaviors Following the 3-Month Intervention.

Variable Group & Assessment	Pre-Intervention n (%)	Post-Intervention n (%)	*p*-Value *
**Mealtime Characteristics**			
Holding food in the mouth			
Very long (≥5 min)	3 (5.4)	0 (0.0)	**<0.001**
Fairly long (3–4 min)	17 (30.4)	3 (5.4)	
Relatively long (1–2 min)	12 (21.4)	18 (32.1)	
Does not hold food	24 (42.9)	35 (62.5)	
Total meal duration			
>60 min	3 (5.4)	1 (1.8)	**<0.001**
45–60 min	24 (42.9)	13 (23.2)	
30–45 min	14 (25.0)	24 (42.9)	
<30 min	15 (26.8)	18 (32.1)	
Mealtime progression			
Holds from start to end	3 (5.4)	1 (1.8)	**<0.001**
Holds after a few bites	12 (21.4)	2 (3.6)	
Holds after half a meal	20 (35.7)	15 (26.8)	
Rapid from start to end	21 (37.5)	38 (67.9)	
Food quantity vs. peers			
Very little (<30%)	0 (0.0)	0 (0.0)	**<0.001**
Fairly little (30–50%)	0 (0.0)	0 (0.0)	
Relatively little (>50%)	25 (44.6)	8 (14.3)	
Normal/High amount	31 (55.4)	48 (85.7)	
**Eating Behaviors**			
Food pickiness			
High (≤3 items)	3 (5.4)	2 (3.6)	**<0.001**
Selective (4–5 items)	28 (50.0)	15 (26.8)	
Varied (6–10 items)	23 (41.1)	33 (58.9)	
All food types	2 (3.6)	6 (10.7)	
Sensory-based refusal			
Always	3 (5.4)	0 (0.0)	**<0.001**
Frequently	24 (42.9)	7 (12.5)	
Occasionally	26 (46.4)	31 (55.4)	
Never	3 (5.4)	18 (32.1)	
Oppositional behaviors			
Very frequent	0 (0.0)	0 (0.0)	**<0.001**
Fairly frequent	14 (25.0)	2 (3.6)	
Occasionally	32 (57.1)	26 (46.4)	
Never	10 (17.9)	28 (50.0)	
Avoidance behaviors			
Very frequent	0 (0.0)	0 (0.0)	**<0.001**
Fairly frequent	12 (21.4)	2 (3.6)	
Occasionally	32 (57.1)	22 (39.3)	
Never	12 (21.4)	32 (57.1)	

* Note: Wilcoxon signed-rank test.

**Table 4:** Changes in Micronutrient and Hematological Indicators.

Biochemical Parameters	Pre-Intervention Mean (SD)	Post-Intervention Mean (SD)	Mean Difference (95% CI)	*p*-Value
Albumin (g/L)	42.76 (3.35)	43.07 (3.72)	0.31 (−0.30–0.93)	0.43
Ferritin (ng/mL)	31.74 (19.03)	34.79 (16.85)	3.05 (0.57–5.54)	**0.001**
Zinc (µmol/L)	10.29 (3.64)	11.72 (2.78)	1.44 (0.67–2.20)	**0.001**
Hemoglobin (g/L)	122.73 (9.30)	124.77 (7.75)	2.04 (0.83–3.24)	**0.002**

**Table 5:** Exploratory Regression Analysis of Anthropometric Changes during the intervention period (*n* = 56).

Outcome & Predictor Variables	Estimate (β)	95% CI	*p*-Value
*Model 1: Change in Height-for-Age (Δ HAZ) ^a^*			
Baseline HAZ	−0.075	(−0.133–−0.017)	**0.013**
Age (months)	0.003	(0.000–0.006)	**0.040**
Δ Behavior Score (0–10)	0.034	(−0.094–0.025)	0.25
Δ Protein Intake (g/day)	−0.004	(−0.013–0.005)	0.35
*Model 2: Change in Weight-for-Age (Δ WAZ) ^b^*			
Baseline WAZ	−0.186	(−0.249–−0.124)	**<0.001**
Age (months)	−0.003	(−0.005–0.001)	0.18
Δ Behavior Score (0–10)	0.030	(−0.027–0.102)	0.25
Δ Protein Intake (g/day)	−0.007	(−0.017–0.003)	0.16
Δ Serum Zinc (µ mol/L)	−0.010	(−0.032–0.012)	0.38

Note: ^a^ Model 1 Diagnostics: F = 2.66, *p* = 0.040, R^2^ = 0.175, Adjusted R^2^ = 0.111. ^b^ Model 2 Diagnostics: F = 11.43, *p* < 0.001, R^2^ = 0.533, Adjusted R^2^ = 0.487. Δ indicates the absolute change from baseline to the post-intervention endpoint.

## Data Availability

The datasets generated and/or analyzed during the current study are not publicly available due to participant privacy, but are available from the corresponding author on reasonable request.

## Appendix A

**Table A1:** Composition of the Multi-nutrient Supplement (AnniBaby Multi) per 20 mL Dose.

Ingredient	Amount per 20 mL
**Vitamins**	
Vitamin C	90 mg
Vitamin PP (Niacin)	20 mg
Vitamin B5 (Pantothenic Acid)	12 mg
Vitamin B1 (Thiamine)	2 mg
Vitamin B2 (Riboflavin)	2 mg
Vitamin B6 (Pyridoxine)	2 mg
Folic Acid	400 mcg
Biotin	100 mcg
Vitamin K2	20 mcg
Vitamin D3	10 mcg (400 IU)
Vitamin B12	1.8 mcg
**Minerals and Trace Elements**	
Potassium	600 mg
Zinc	6 mg
Manganese	4 mg
Copper	600 mcg
Iodine	120 mcg
Selenium	40 mcg
**Amino Acids and Others**	
L-Lysine	100 mg
L-Leucine	70 mg
L-Isoleucine	70 mg
Betacarotene	800 mcg
